# Serum Levels of S100b and NSE Proteins in Patients with Non-Transfusion-Dependent Thalassemia as Biomarkers of Brain Ischemia and Cerebral Vasculopathy

**DOI:** 10.3390/ijms18122724

**Published:** 2017-12-15

**Authors:** Aikaterini Kanavaki, Konstantinos Spengos, Maria Moraki, Polyxeni Delaporta, Catherine Kariyannis, Ioannis Papassotiriou, Antonis Kattamis

**Affiliations:** 1 First Department of Pediatrics, National and Kapodistrian University of Athens, 11527 Athens, Greece; marmoraki@gmail.com (M.M.); polyxenidelaporta@yahoo.gr (P.D.); ankatt@med.uoa.gr (A.K.); 2 First Department of Neurology, “Eginition” Hospital, National and Kapodistrian University of Athens, 11528 Athens, Greece; kspengos@med.uoa.gr; 3 Department of Clinical Biochemistry, “Aghia Sophia” Children’s Hospital, Goudi, 11527 Athens, Greece; kariyiannis@paidon-agiasofia.gr (C.K.); biochem@paidon-agiasofia.gr (I.P.)

**Keywords:** non-transfusion-dependent thalassemia (NTDT), Transcranial Doppler (TCD), stroke, silent infarct, vasculopathy, lactate dehydrogenase (LDH), neuron specific enolase (NSE), S100 calcium-binding protein B (S100B)

## Abstract

Patients with non-transfusion-dependent thalassemia (NTDT) are at risk of developing brain ischemia. Transcranial Doppler (TCD) has been established as a useful screening tool of cerebrovascular disease in patients with sickle cell disease. Proteins neuron specific enolase (NSE) and S100B are biomarkers that reflect CNS injury. The purpose of this study is to evaluate cerebral vessel vasculopathy and brain damage in NTDT patients using non-invasive methods as TCD and measurement serum levels of NSE and S100B. We included in our study 30 patients with NTDT, aged between 8 and 62 years old (mean: 29.4, median: 32) who presented in our Unit for regular follow-up. We performed in all patients a non-imaging TCD examination and have measured serum S100, NSE and lactate dehydrogenase (LDH) levels. We investigated the possible correlation between TCD results and S100B, NSE and LDH levels as well as between NSE-LDH and S100B-LDH levels by regression analysis. We found a statistically significant relationship for both NSE, S100B with LDH. We also found a statistically significant relationship for S100B and time-averaged mean velocity (TAMV)/peak velocity of left middle cerebral artery (MCA), NSE and pulsatility index (PI)/resistive index (RI) of the left posterior cerebral artery (PCA). TCD results correlated with biomarkers for brain ischemia. This finding enhances the role of TCD as a screening tool for brain ischemia in patients with NTDT.

## 1. Introduction

The term non-transfusion-dependent thalassemia (NTDT) refers to patients with thalassemia but without need of regular transfusions even though they may require occasional or even frequent transfusions in certain clinical conditions and for defined periods of time. It includes: β-thalassemia intermedia, α-thalassemia intermedia and hemoglobin E/β-thalassemia [1]. NTDT was considered in the past as a mild condition. However, recent studies show that patients with NTDT are prone to suffer thromboembolic events. Patients who have been splenectomized are more prone to thromboembolism. [2].

Patients with NTDT have 4.38 times a greater risk of developing a thromboembolic event compared to β-thalassemia major (βΤM) patients as reported in a recent study on 8860 patients with β-thalassemia major and intermedia [3]. Thromboembolism can manifest as deep venous thrombosis, pulmonary embolism, portal vein thrombosis, superficial thrombophlebitis and ischemic strokes. Ischemic strokes consist a major complication in patients with NTDT and can be divided in two categories: overt strokes and silent infarcts. In contrast to an overt stroke a silent infarct is not associated to a neurologic abnormality and cannot be detected on clinical examination [4].

Although overt strokes are more frequent in patients with βΤM than in those with β NTDT, recent studies have shown a high incidence of clinically silent cerebral ischemic events (CIEs) in splenectomized patients with β NTDT [5,6,7,8,9]. The underline pathophysiology of ischemic strokes in these patients has not been clarified: large vessel disease, microangiopathy and venous thrombosis have been suggested as possible pathophysiologic mechanisms [2,10,11].

Transcranial Doppler (TCD) has been established as a useful tool in screening cerebrovascular disease in sickle cell disease (SCD) as demonstrated in a large randomized clinical trial performed in the 1990s, the STOP trial (stroke prevention trial in sickle cell anemia). The implementation of the STOP criteria has led in a significant decrease of ischemic strokes in SCD patients [12,13,14]. Little is reported in literature about the use of TCD in NTDT patients.

Neuron specific enolase (NSE) and S100 calcium-binding protein B (S100B) are considered as biomarkers that reflect CNS injury [15]. More specifically, increased levels of NSE and S100B have been reported in patients with ischemic stroke, brain hypoxia, head trauma and other pathologies resulting to glial cell or neuronal damage [16,17,18,19,20,21,22].

High blood levels of lactate dehydrogenase (LDH) have been related in literature with vasculopathy in patients with sickle cell disease [23].

The purpose of this study is to evaluate cerebral vessel vasculopathy and brain damage in patients with NTDT using non-invasive methods as TCD and measurement of NSE, S100B and LDH in blood serum.

## 2. Results

Hemoglobin (Hb) levels ranged between 7.1 and 13.5 g/dL (mean: 9.4, median: 9.2), hematocrit levels (Ht) ranged between 25.3 and 44.8 (mean: 32.8, median: 31.6) % and platelet count (PLT) ranged between 212 and 1025 (mean: 513, median: 457.5) × 10^3^/mm^3^.

NSE values ranged between 3 and 17.38 (mean: 9.9, median: 9.7, SD = 4.36) ng/mL and S100B values between 0.007 and 0.133 ng/mL (mean: 0.049, median: 0.043, SD = 0.03) ng/mL in patient group. In the control group, NSE values ranged between 2.86 and 17.1 (mean: 9.98, median: 9.98, SD = 4.51) ng/mL and S100B values between 0.027 and 0.104 ng/mL (mean: 0.056, median: 0.055, SD = 0.02) ng/mL. There was no statistically significant difference of NSE and S100B serum levels between patients and normal controls (*p* = 0.888 and *p* = 0.167 respectively) (Table 1).

LDH levels ranged between 188 and 847 U/L (mean: 439 U/L, median 396 U/L). The mean LDH level was higher than normal ranges (normal ranges: 140–280 U/L).

As far as TCD is being concerned, all patients had time-averaged mean velocity (TAMV) values < 170 cm/s (TAMV value considered as conditional according to the STOP criteria for SCD) [24].

**Figure 1:**
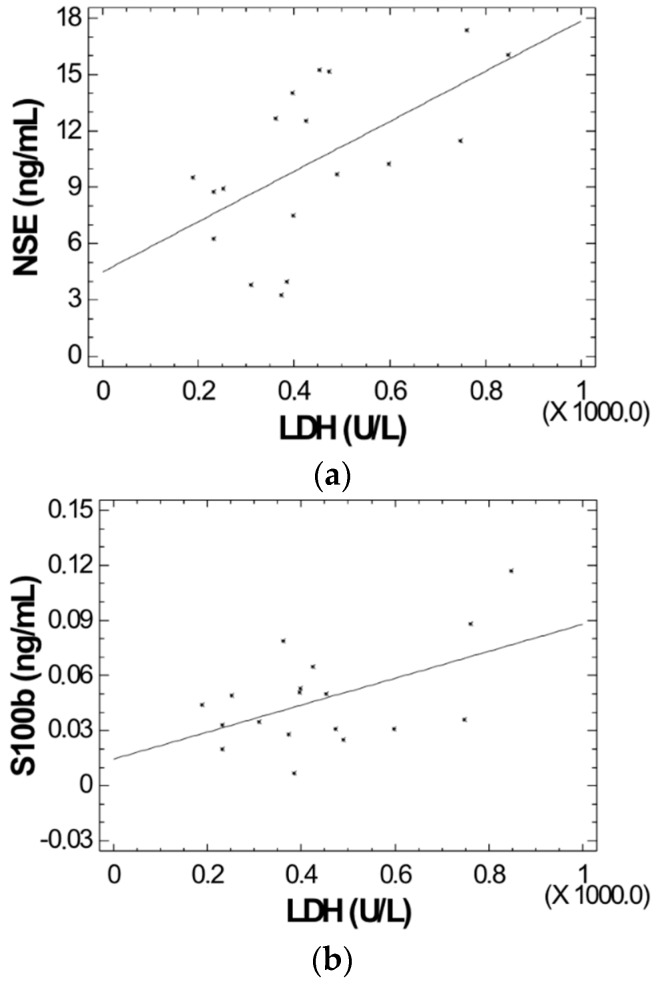
(**a**) Correlation of lactate dehydrogenase (LDH) levels and neuron specific enolase (NSE) concentrations in patients with non-transfusion-dependent thalassemia (NTDT) (*r* = 0.586, *p* < 0.01); (**b**) Correlation of LDH levels and S100b protein concentrations in patients with NTDT (*r* = 0.520, *p* = 0.02).

For 18 patients where LDH were measured, we compared LDH values to NSE and S100B serum levels using regression analysis. We found a statistically significant relationship for both NSE and S100B with LDH (*p* < 0.01 and *p* = 0.02 respectively) (Figure 1a,b)

**Figure 2:**
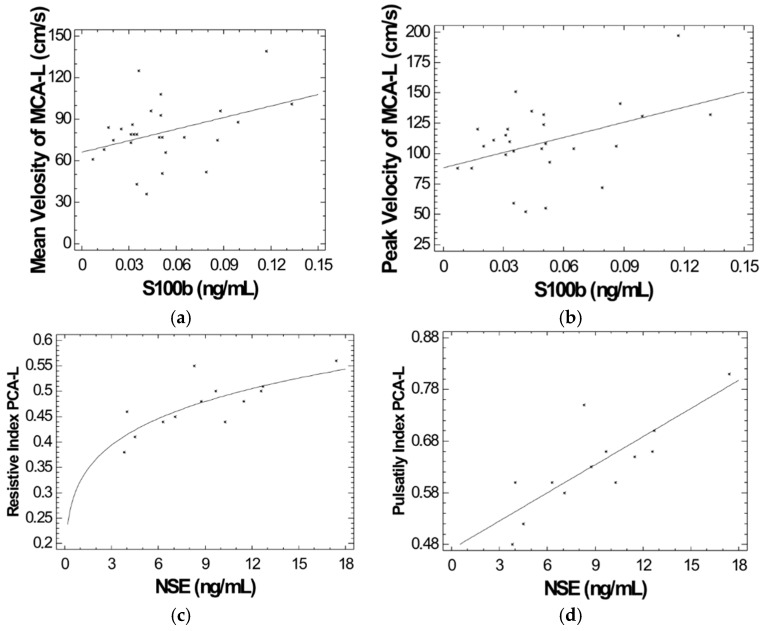
(**a**) Correlation of S100b protein levels and time-averaged mean velocity (TAMV) of left middle cerebral artery (MCA-L) in patients with NTDT (*r* = 0.384, *p* < 0.05); (**b**) Correlation of S100b protein levels and peak velocity of left middle cerebral artery (MCA-L) patients with NTDT (*r* = 0.421, *p* = 0.02); (**c**) Correlation of NSE levels and resistive index (RI) of left posterior cerebral artery (PCA-L) in patients with NTDT (*r* = 0.761, *p* = 0.002); (**d**) Correlation of NSE levels and pulsatility index of left posterior cerebral artery (PCA-L) in patients with NTDT (*r* = 0.815, *p* < 0.001).

We also found a statistically significant relationship between the values of S100B and those of TAMV (*r* = 0.384, *p* < 0.05) and Peak Velocity (*r* = 0.421, *p* = 0.02) of the left middle cerebral artery (MCA-L) and between the values of NSE and resistive index (RI) (*r* = 0.761, *p* = 0.002) and pulsatility index (PI) (*r* = 0.815, *p* < 0.001) of the (PCA-L) (Figure 2a–d).

There was no statistically significant relationship between the values of LDH and the parameters of TCD.

## 3. Discussion

Thromboembolism consist a major complication in patients with NTDT and can manifest as cerebral ischemia, either overt strokes or silent cerebral infarcts [5,6,7,8,9]. The underline pathophysiology that has been suggested is large vessel disease, microangiopathy and venous thrombosis [2,10,11].

S100B is considered to be a biomarker reflecting CNS injury. This protein is a member of a family of Ca^+^-binding proteins and is expressed in specific cell types as astrocytes, maturing oligondendrocytes, neural progenitor cells, adipocytes, melanocytes skeletal myofibers and myoblasts [25]. It is found in abundance in the nervous system, mostly in astrocytes and several neuronal populations. At low levels S100B acts as a neurotrophic factor whereas in high levels it can act in the opposite way [16]. Increased serum levels of this protein can be found in patients with cerebrovascular disease, brain hypoxia due to cardiovascular arrest, cerebral trauma, but also in patients with schizophrenia, epilepsy or Alzheimer’s disease [15,16,19,21,22].

Neuron specific enolase (NSE) is a dimeric isoenzyme of the glycolytic enzyme enolase. It is localized mainly within neurons and cells of neuroendocrine origin and is considered to be a biomarker for neuronal loss [20]. Increased serum levels of NSE have been reported in patients with multiple sclerosis, CNS tumors, head trauma, stroke and brain hypoxia [17,18].

The role of LDH as an index of hemolysis in SCD patients is already known. High levels of LDH are related to increased resistance to nitric oxide (NO), endothelial dysfunction and vasculopathy [23]. Studies in literature have shown relationship between the levels LDH and the parameters of TCD in SCD patients. In a study from Bernaudin et al., LDH levels is an independent prognostic factor of high TAMV values in TCD, supporting that cerebral microangiopathy can be related to hemolysis and NO levels [26].

We studied the relationship of NSE (biomarker of neuronal loss) and S100B (biomarker of glial destruction) with TCD parameters (index of cerebrovascular disease in patients with NTDT. We also looked for a relationship between NSE, S100B and LDH serum levels. We found no relationship between LDH and TCD parameters.

We found, however, a statistically significant relationship between the serum levels of NSE and S100B and those of LDH that is between biomarkers of neuronal and glial cells destruction and an index of hemolysis. This finding is in accordance with the aforementioned study of Bernaudin et al., supporting a possible pathophysiologic relationship between neuronal and glial cell destruction, hemolysis and alterations in NO levels in these patients.

We found a statistically significant relationship between the levels of S100 and the values of TAMV and peak velocity of left MCA and the levels of NSE and RI, PI of the left PCA. TCD values reflect the hemodynamics of brain circulation and it is well known that TCD is used in SCD patients as an effective screening tool in predicting the risk of stroke in these patients [12,24]. We found a statistically significant relationship between the values of TCD and those of NSE and S100B in our patients that is between an index of cerebral vascular circulation/hemodynamics and biomarkers of neuronal and glial cells destruction respectively. We have no explanation, however, for the reason that this relationship was statistically significant only for the left MCA and PCA and not for all the examined vessels.

A major limitation of our study is the small sample size. Further investigations are needed including a larger patient group in order to validate these results.

In summary, our study enhances the role of TCD as a screening tool for evaluation of brain ischemia risk in patients with NTDT. It also supports the hypothesis that hemolysis plays an important role in the pathophysiology of brain ischemia in these patients.

## 4. Material and Methods

This was a prospective study conducted during an 18-month period at the Hematology Unit and the Department of Neurology of our tertiary university hospitals. The Ethical Committee of “Aghia Sophia“ Children’s hospital approved the study (Approval Number: 17550; 5 August 2011), respecting the Declaration of Helsinki. All patients have given informed consent to participate in the study. 

We included in the study 35 patients (22 M:13 F) with the diagnosis of NTDT, aged 13–63 years old (mean 33.8, median 34) who were regularly followed in our unit. Patients with known thromboembolic events, cerebrovascular or cardiovascular ischemic attacks, hypertension, diabetes mellitus, under anticoagulant therapy or with an inadequate window for TCD were excluded from the study. The final study group included 30 patients (21 M: 9F), aged 8–62 years old (mean: 29.4, median: 32).

Diagnosis of NTDT was made according to the criteria for thalassemia intermedia described by Kattamis et al. [27]. For all patients, laboratory data included hemoglobin, hematocrit and ferritin levels, and platelet count. All patients were transfusion-independent during this study and had no recent history of transfusion.

All TCD examinations were performed by an experienced investigator (KS) using a non-imaging TCD device (RIMED Inc., Raanana, Israel) with a 2 MHz transducer. The anterior (ACA), middle (MCA) and posterior (PCA) cerebral arteries were insonated through a temporal window approach and the basilar artery (BA) through a sub-occipital window. For each vessel, the highest value of the time-averaged mean velocity (TAMV) was registered, as well as the peak velocity, resistive index (RI, RI = (Peak Systolic velocity-End diastolic velocity)/Peak Systolic velocity) and pulsatility index (PI, PI = (Peak Systolic velocity-End diastolic velocity)/Mean velocity) [28]. We evaluated TAMV values according to the STOP criteria. According to the criteria established in this trial, the TCD parameter used for discriminating high-risk from low-risk SCD patients for ischemic stroke is the time-averaged mean velocity (TAMV) measured in the middle cerebral artery (MCA), distal internal carotid (dICA) and its bifurcation (BIF). A TAMV value between 170 and 200 cm/s is considered to be conditional and a TAMV ≥ 200 cm/s as abnormal. [14,24]

The serum levels of S100B protein was determined by chemiluminescence using a commercial available assay provided from the Sangtec Medical AB, Bromma, Sweden. The measurements were performed using the LIAISON random access analyzer (DiaSorin, Saluggia, Italy). According to the manufacturer the minimum detectable concentration of the assay is 0.02 ng/mL, and the intra-and inter-assay coefficients of variation for S100B were less than 3.8% and 5.7%, respectively.

The serum levels of NSE were determined a commercial with and electrochemiluminescence immunoassay using the fully automated electrochemiluminescence system Elecysys 2010 (Roche Diagnostics, Mannheim, Germany). According to the manufacturer the minimum detectable concentration of the assay is 0.015 ng/mL and the intra-and inter-assay coefficients of variation for NSE were less than 3.2% and 5.0%, respectively.

Serum levels of NSE and S100B of our patients were compared to those of 26 age-sex matched controls.

In 18 patients LDH levels were also measured and compared to S100B and NSE levels as well as to TCD results. LDH levels were compared to normal values (normal ranges 140–280 U/L).

We investigated the possible correlation between TCD results and S100B, NSE and LDH levels as well as between NSE-LDH and S100B-LDH levels by regression analysis. For statistical analysis, free online tools such as Statgraphics Stratus (Available online: http://www.statpoint.net/About.aspx) and Social Science Statistics (Available online: http://www.socscistatistics.com) were used.

## Figures and Tables

**Table 1:** Neuron specific enolase (NSE) and S100 B serum levels in patients and control group (ng/mL).

Patients	NSE (ng/mL)	S100B (ng/mL)	Controls	NSE (ng/mL)	S100B (ng/mL)
P1	4.5	0.014	C1	6.94	0.104
P2	7.07	0.035	C2	4.45	0.059
P3	8.75	0.033	C3	2.86	0.048
P4	16.07	0.117	C4	2.91	0.059
P5	15.25	0.050	C5	7.49	0.027
P6	17.38	0.088	C6	7.98	0.046
P7	9.68	0.025	C7	5.69	0.042
P8	3.00	0.05	C8	6.12	0.032
P9	3.7	0.017	C9	8.67	0.050
P10	4.00	0.007	C10	12.77	0.075
P11	3.81	0.035	C11	14.55	0.051
P12	3.27	0.028	C12	4.44	0.074
P13	7.50	0.053	C13	13.95	0.049
P14	14.03	0.051	C14	15.72	0.059
P15	12.54	0.065	C15	15.54	0.070
P16	15.1	0.041	C16	9.74	0.030
P17	5.13	0.086	C17	15.82	0.027
P18	7.48	0.099	C18	13.91	0.068
P19	11.3	0.051	C19	13.35	0.027
P20	11.48	0.049	C20	14.96	0.082
P21	8.94	0.031	C21	6.48	0.027
P22	10.26	0.044	C22	12.71	0.053
P23	15.15	0.133	C23	17.10	0.082
P24	9.52	0.079	C24	10.22	0.075
P25	14.56	0.020	C25	4.70	0.056
P26	12.68	0.032	C26	10.38	0.083
P27	12.57	0.032			
P28	6.28	0.036			
P29	14.60	0.031			
P30	8.3	0.051			
